# Special Issue “Circulating Non-Coding RNAs as Diagnostic and Prognostic Markers of Human Diseases, 2nd Edition”

**DOI:** 10.3390/ijms26199449

**Published:** 2025-09-27

**Authors:** Christos Papaneophytou, Kyriacos Felekkis

**Affiliations:** 1 Department of Life Sciences, School of Life and Health Sciences, University of Nicosia, Nicosia 2417, Cyprus; felekkis.k@unic.ac.cy; 2 Non-Coding RNA Research Laboratory, School of Life and Health Sciences, University of Nicosia, Nicosia 2417, Cyprus

The dynamic transcriptomic landscape of the human genome, comprising both coding and non-coding RNA (ncRNA) transcripts, serves as a molecular fingerprint of cellular and tissue-specific physiological states [1]. Among these, ncRNAs have emerged as key “architects” of eukaryotic genomic complexity, orchestrating an additional layer of regulatory control that enables the integration of intricate gene expression programs at the cellular level [2].

In recent years, the field of molecular diagnostics has undergone a transformative shift with the recognition of ncRNAs, including microRNAs (miRNAs or miRs), long non-coding RNAs (lncRNAs), circular RNAs (circRNAs), and PIWI-interacting RNAs (piRNAs) as potent biomarkers. Their abundance, stability, and detectability in biological fluids such as blood and urine make them ideal candidates for non-invasive diagnostic and prognostic applications. These molecules have demonstrated considerable promise across a broad spectrum of diseases, including cancer, cardiovascular disorders, autoimmune conditions, and neurodegenerative diseases, offering valuable insights into early detection, therapeutic response, and disease recurrence [3].

Beyond their diagnostic utility, ncRNAs are increasingly recognized for their roles in intercellular communication. Through extracellular vesicle-mediated transfer, ncRNAs can influence gene expression in recipient cells, with profound implications for cancer progression, metastasis, and therapy resistance. This emerging understanding opens new avenues for therapeutic targeting and personalized medicine [4].

Despite early challenges related to variability and standardization, recent technological and analytical advances have significantly advanced the clinical integration of ncRNA-based biomarkers. This second edition of our Special Issue extends the foundational work of its predecessor, presenting novel research and critical reviews spanning oncology, neurology, autoimmune diseases, and reproductive health. Collectively, these contributions underscore the multifaceted roles of ncRNAs in human health and disease. Below is an overview of the original and review articles published in this Special Issue.

The first article by Nunez et al. (contribution 1) compares serum and plasma to determine which is a more suitable source for detecting circulating miRNAs (c-miRNAs) in sudden sensorineural hearing loss (SSNHL), an acute idiopathic hearing loss developing within 72 h. Building on their earlier identification of eight differentially expressed miRNAs (miR-128-3p, miR-132-3p, miR-375-3p, miR-590-5p, miR-30a-3p, miR-140-3p, miR-186-5p, and miR-195-5p) in SSNHL serum samples, they compared expression levels in paired serum and plasma samples from 17 patients. The results revealed no statistically significant differences between the two sample types. These results indicate that both serum and plasma are equally suitable for studying these SSNHL-associated miRNAs, supporting their potential use in non-invasive diagnostics for inner ear disorders when samples are processed promptly after collection.

In the second article, Miyano et al. (contribution 2) sought to verify the reproducibility of plasma miRNA-based stratification of patients with schizophrenia previously reported by their group [5], and explore symptom-related pathophysiological pathways. For this, they quantified 376 plasma miRNAs from 70 patients, alongside Positive and Negative Syndrome Scale (PANSS) and Brief Assessment of Cognition in Schizophrenia (BACS) scores. Hierarchical clustering of miRNA profiles reproduced three patient subgroups, characterized by different inflammatory backgrounds, similar to those in their earlier study, highlighting further the robustness of miRNA-based stratification. Multivariate modeling identified optimal miRNA combinations that estimated positive, negative, and cognitive symptom scores, with enrichment analyses linking these miRNAs to inflammation-related pathways (notably NF-κB, IL-1β, IL-6, and TNFα). These findings highlight further the potential of plasma miRNAs as biomarkers for patient stratification and reveal molecular mechanisms of symptom heterogeneity in schizophrenia.

The third article by Piccinno et al. (contribution 3) investigates the potential of three c-miRNAs, namely miR-23b-3p, miR-30e-3p, and miR-205-5p, as predictive biomarkers in 40 patients with advanced gastric cancer receiving second-line therapy with Ramucirumab plus Paclitaxel, stratified by their progression-free survival (PFS). Patients with longer PFS exhibited a progressive and significant decrease in the levels of these miRNAs to minimal values over the course of treatment. Notably, baseline miR-205-5p levels were inversely correlated with angiopoietin-2 concentrations. Higher baseline miR-205-5p was associated with a protective effect and more prolonged overall survival. The results of this study suggest that miR-23b-3p, miR-30e-3p, and miR-205-5p may serve as non-invasive biomarkers for predicting response to anti-angiogenic therapy in advanced gastric cancer.

The work by Choi et al. (contribution 4) is a high-throughput transcriptomic profiling study of eutopic endometrial tissues from women with endometriosis (EMS) and controls (CT) during the proliferative (P) and secretory (S) phases of the menstrual cycle, using total RNA-Seq. Samples were obtained from 18 controls (pCT = 8; sCT = 13) and 23 EMS patients (pEMS = 13; sEMS = 12) to identify differentially expressed long non-coding RNAs (DElncRNAs) and mRNAs (DEmRNAs). In EMS tissues during the P-phase, genes related to cell adhesion, angiogenesis, TNF response, and endothelial cell proliferation showed altered expression. NONHSAG019742.2 and NONHSAT120701.2 were upregulated, whereas NONHSAG048398.2 and NONHSAG016560.2 were downregulated. Neighboring gene analysis showed concordant changes: HSD11B2 was increased, and THBS1 was decreased. ELP3, a regulator of cell migration, was upregulated, and NR4A1, involved in endothelial proliferation, was downregulated. In S-phase EMS tissues, NONHSAT000959.2, NONHSAT203423.1, and NONHSAG053769.2 were upregulated, while NONHSAG012105.2 and NONHSAG020839.2 were downregulated, with matching changes in neighboring genes GPX3 (increased) and SHISA6 (decreased). LAMB3 and HIF1A, linked to cancer pathways, were increased, whereas PAM, associated with hypoxia response, was reduced. Together, these findings highlight phase-specific transcriptomic alterations in EMS and suggest potential lncRNA–mRNA biomarker pairs for diagnosis or prognosis. However, the relatively small cohort size may limit generalizability, underscoring the need for validation in larger studies.

The study by Tovar-Cuevas et al. (contribution 5) is a bioinformatics analysis of c-miRNAs in chronic migraine to identify potential regulators of genes involved in inflammation, neovascularization, and pain-related pathways. Plasma samples from female patients with clinically diagnosed chronic migraine (interictal phase, no recent medication use) were analyzed using multiple bioinformatic tools, including miRBase, TargetScan, miRPath, Tissue Atlas, and miR2Disease. Of the 192 miRNAs screened, 63 were not expressed; expression tertiles ranked the remainder. Five miRNAs including miR-197, miR-101, miR-92a, miR-375, and miR-146b were over-expressed, while five were under-expressed—miR-133a/b, miR-134, miR-195, and miR-340. Pathway analysis linked these miRNAs to processes such as neuroinflammation, vascular development, nociceptive pain signaling, and drug resistance. The authors highlighted that these miRNAs, many of which have not previously been reported in chronic migraines, may serve as candidate biomarkers for disease characterization and could help elucidate molecular mechanisms underlying migraine pathophysiology.

The article by Mikulski et al. (contribution 6) evaluates the predictive potential of five serum miRNAs, namely miR-122-5p, miR-122-3p, miR-15b-5p, miR-99b-5p, and miR-125a-5p, for early hepatic toxicity in 66 patients undergoing autologous hematopoietic stem cell transplantation (ASCT), including 50 with multiple myeloma and 16 with lymphoma. Using multivariate logistic regression adjusted for age, sex, and conditioning regimen, two miRNAs (miR-122-5p and miR-125a-5p) emerged as independent predictors of liver injury within 14 days post-transplant. Notably, elevated miR-122-5p was associated with increased risk, while higher miR-125a-5p was protective. These findings suggest that pre-transplant serum miR-122-5p may serve as a risk biomarker for ASCT-related hepatotoxicity, whereas miR-125a-5p could indicate reduced susceptibility to liver injury.

In the seventh paper published in this Special Issue, Wosniaki et al. (contribution 7) evaluate 35 c-miRNAs in plasma samples from patients with chronic myeloid leukemia (CML) at single time points and in follow-up analyses, aiming to identify potential miRNA-based biomarkers linked to disease status and *BCR::ABL1* transcript levels. Among the miRNAs tested, miR-7-5p was significantly upregulated in patients with high *BCR::ABL1* expression compared to healthy controls. In treatment-free remission follow-up, 3 of 4 patients maintained stable miR-7-5p expression, suggesting a possible association between stable levels and disease control. Two patients showed unstable patterns. For instance, one exhibited increased miR-7-5p expression at specific follow-up points despite undetectable *BCR::ABL1*, indicating that other factors may influence its regulation. Overall, the findings suggest that miR-7-5p may be related to CML in a non-specific manner, primarily through its association with *BCR::ABL1* transcript levels, but its variability across patients highlights the need for further investigation before clinical application.

The study by Patel et al. (contribution 8) examines the potential of serum lncRNAs as biomarkers for repetitive mild traumatic brain injury (rmTBI) in U.S. veterans. Samples were obtained from the Long-Term Impact of Military-Relevant Brain-Injury Consortium Chronic Effects of Neurotrauma Consortium (LIMBIC CENC) repository, from participants either unexposed to TBI or with a history of rmTBI. Initial screening identified four lncRNAs—MALAT1, GAS5, NEAT1, and VLDLR-AS1—consistently present in all samples. Among these, VLDLR-AS1 levels were significantly lower in individuals with rmTBI compared to those with no lifetime TBI. Secondary analysis of LIMBIC CENC clinical data showed that VLDLR-AS1 and MALAT1 levels correlated with depression severity, but not with post-traumatic stress disorder (PTSD) or overall post-concussive symptom burden. The authors conclude that reduced serum VLDLR-AS1 represents a promising biomarker for chronic rmTBI—detectable years after injury—and may also be linked to depression. These findings support the concept that rmTBI leaves persistent molecular signatures that could aid in long-term diagnosis and monitoring.

Cieśla et al.’s article (contribution 9) investigates the association between selected circRNAs and disease activity in rheumatoid arthritis (RA). A total of 71 participants were enrolled: 45 RA patients and 26 healthy controls (HCs). Within the RA group, 24 patients had high disease activity (DAS-28-ESR > 5.1) and 21 were in remission (DAS-28-ESR ≤ 2.6). Plasma analysis showed that circ_0005567 levels were significantly elevated in RA patients compared to HCs. Patients with high disease activity had higher circ_0005567 concentrations than controls. Functional studies in the SW982 synovial sarcoma cell line revealed that circ_0005567 expression was inversely associated with miR-194-5p levels and linked to increased expression of mRNAs potentially related to cell proliferation. The authors propose that circ_0005567 may act as a molecular sponge for miR-194-5p, thereby reducing its availability. In silico analysis also suggested miR-492 as another potential target. The findings suggest that decreased circ_0005567 expression and increased miR-194-5p may contribute to RA pathogenesis. The observed relationship between circ_0005567 and miR-194-5p highlights a possible regulatory axis with relevance to inflammation and joint pathology.

The review article by Yan and Wang (contribution 10) provides a comprehensive overview of the biology and functions of ncRNAs, including miRNAs, circRNAs, and lncRNAs—in the regulation of spermatogenesis. The authors discuss how these ncRNAs influence germ cell development, meiosis, and sperm maturation, as well as their roles in testicular somatic cells such as Sertoli and Leydig cells. The review highlights correlations between aberrant ncRNA expression and male infertility and evaluates the potential of specific ncRNAs as non-invasive biomarkers for sperm quality and fertility. The authors also outline current gaps in functional annotation, emphasize the need for validation in human studies, and explore the prospects of ncRNA-based diagnostics and therapeutics for male infertility.

In the second review published in this Special Issue, Seyhan (contribution 11) provides a comprehensive overview of the current literature on circulating biomarkers as potential minimally invasive or non-invasive tools for guiding glioma treatment, with a particular emphasis on glioblastoma (GBM). The author outlines the limitations of current diagnostic approaches, which typically involve neuroimaging techniques such as MRI or CT scans followed by surgical resection or biopsy of tumor tissue, and highlights the promise of liquid biopsies in overcoming these challenges. In GBM, the successful application of liquid biopsy depends on the ability of tumor-derived material to cross the blood–brain barrier (BBB). This selective interface regulates the exchange of molecules between the bloodstream and the brain. Liquid biopsies can detect and quantify a range of circulating biomarkers, including circulating tumor cells (CTCs), extracellular vesicles (EVs) carrying nucleic acids and proteins, circulating tumor DNA (ctDNA), and miRNAs. These analytes include tumor-related data, such as mutational profiles and molecular content, which can be accessed through minimally invasive sampling methods. This approach enables disease monitoring, evaluation of treatment response, and may allow for earlier detection of recurrence.

Finally, in the third review, Felekkis and Papaneophytou (contribution 12) examine the potential of integrating three well-studied classes of circulating biomarkers—c-miRNAs, cell-free DNAs (cfDNAs), and proteins—detected in blood and other biofluids, to improve the accuracy and efficacy of disease detection and monitoring. The authors provide an overview of the unique characteristics, advantages, and limitations of each biomarker class, emphasizing the superior stability and specificity of c-miRNAs, the genetic insights offered by cfDNAs, and the functional information derived from circulating proteins. They highlight how a multi-analyte approach can overcome the limitations of single-marker diagnostics, as the combined analysis of these complementary biomarkers can markedly enhance diagnostic sensitivity and specificity. The review also discusses the challenges that must be addressed before such integrated liquid biopsy strategies can be translated into clinical practice, including the need for standardized protocols, validation in large patient cohorts, and cost-effective analytical platforms.

Together, the articles in this Special Issue highlight the significant potential of ncRNAs in the prognosis, diagnosis, and monitoring of disease progression across a wide range of conditions beyond cancer. Combining ncRNAs with other circulating biomarkers—such as cfDNA, proteins, metabolites, and extracellular vesicles—provides a promising pathway toward more accurate, sensitive, and comprehensive diagnostic approaches.

Looking ahead, the field will benefit from large-scale, multicenter validation studies, standardized analytical protocols, and longitudinal research to track ncRNA dynamics over time. Multi-omics approaches and mechanistic studies will be essential to uncover the biological roles of ncRNAs and translate them into actionable clinical tools. By bridging discovery science with clinical application, future research can unlock the full potential of ncRNAs as part of next-generation precision medicine.

## Data Availability

No new data was created in this study.
